# A small stem-loop-forming region within the 3′-UTR of a nonpolyadenylated LCMV mRNA promotes translation

**DOI:** 10.1016/j.jbc.2022.101576

**Published:** 2022-01-10

**Authors:** Mei Hashizume, Ayako Takashima, Masaharu Iwasaki

**Affiliations:** 1 Laboratory of Emerging Viral Diseases, International Research Center for Infectious Diseases, Research Institute for Microbial Diseases, Osaka University, Suita, Osaka, Japan; 2 Center for Infectious Disease Education and Research (CiDER), Osaka University, Suita, Osaka, Japan

**Keywords:** translation control, RNA-binding protein, mRNA, negative-strand RNA virus, translation regulation, BUNV, Bunyamwera virus, GPC, glycoprotein precursor, IGR, intergenic region, LASV, Lassa virus, LAV, live-attenuated vaccine, LCMV, lymphocytic choriomeningitis virus, LR, loop region, NP, nucleoprotein, PABP, poly(A)-binding protein, PR, proximal region, UTR, untranslated region, vmRNA, viral mRNA

## Abstract

Mammalian arenavirus (mammarenavirus) mRNAs are characterized by 5′-capped and 3′-nonpolyadenylated untranslated regions (UTRs). We previously reported that the nonpolyadenylated 3′-UTR of viral mRNA (vmRNA), which is derived from the noncoding intergenic region (IGR), regulates viral protein levels at the posttranscriptional level. This finding provided the basis for the development of novel live-attenuated vaccines (LAVs) against human pathogenic mammarenaviruses. Detailed information about the roles of specific vmRNA 3′-UTR sequences in controlling translation efficiency will help in understanding the mechanism underlying attenuation by IGR manipulations. Here, we characterize the roles of *cis*-acting mRNA regulatory sequences of a prototypic mammarenavirus, lymphocytic choriomeningitis virus (LCMV), in modulating translational efficiency. Using *in vitro* transcribed RNA mimics encoding a reporter gene, we demonstrate that the 3′-UTR of nucleoprotein (NP) mRNA without a poly(A) tail promotes translation in a poly(A)-binding protein-independent manner. Comparison with the 3′-UTR of glycoprotein precursor mRNA, which is translated less efficiently, revealed that a 10-nucleotide sequence proximal to the NP open reading frame is essential for promoting translation. Modification of this 10-nucleotide sequence also impacted reporter gene expression in recombinant LCMV. Our findings will enable rational design of the 10-nucleotide sequence to further improve our mammarenavirus LAV candidates and to develop a novel LCMV vector capable of controlling foreign gene expression.

Rodent-borne mammalian arenaviruses (mammarenaviruses) include several human pathogenic agents that are responsible for diseases ranging from mild febrile illnesses to life-threatening viral hemorrhagic fever. Lassa virus (LASV) is the most significant agent among mammarenaviruses, and it raises special public health concerns in West African countries (1). LASV infects several hundred thousand individuals yearly, which results in a high number of Lassa fever cases with significant mortality. Moreover, a novel mammarenavirus, Lujo virus, was identified from patients of the recent cluster of hemorrhagic fever cases in Zambia and the Republic of South Africa, which raises concerns about the potential future emergence of novel mammarenaviruses that cause hemorrhagic fever (2, 3). Mounting evidence has also indicated that a globally distributed prototypic mammarenavirus, lymphocytic choriomeningitis virus (LCMV), is an important human pathogen of clinical significance, especially in immunocompromised or pregnant individuals (4, 5, 6, 7). Despite their significant impact on human health, medical countermeasures against human pathogenic mammarenaviruses are limited. There are no licensed vaccines against LASV, and off-label use of ribavirin is currently the most practical approach for treating patients with Lassa fever. However, ribavirin is only partially effective and associated with significant adverse effects (8, 9, 10).

Mammarenaviruses have a bisegmented single-stranded RNA genome that uses an ambisense coding strategy to direct the expression of viral mRNAs from two viral genes arranged in opposite orientations, separated by a noncoding intergenic region (IGR) (11). The S segment RNA encodes the viral nucleoprotein (NP) and the glycoprotein precursor (GPC). The GPC is cotranslationally processed by signal peptidase to generate a stable signal peptide (SSP) and posttranslationally processed by site 1 protease to generate the mature virion surface glycoproteins GP1 and GP2. GP1 and GP2 together with the SSP form the glycoprotein complex, which is responsible for receptor recognition and cell entry. The L segment RNA encodes the viral RNA-dependent RNA polymerase (L) and the matrix RING finger protein (Z). Unlike most cellular mRNAs, mammarenaviral mRNA has a nonpolyadenylated 3′-UTR. Transcription by the L protein is initiated from the 3′-termini of genomic and antigenomic RNAs using a 5′-capped RNA fragment for priming obtained from cellular mRNA by cap snatching, and transcription terminates within the IGR, generating 5′-capped and 3′-nonpolyadenylated viral mRNA (vmRNA) (12, 13, 14, 15). Heterogeneity of the ends of the 3′-UTR indicates that transcription termination is induced by the secondary structure of the IGR, rather than by a specific terminator signal sequence (16). We used an LCMV minigenome system and showed that swapping of the IGRs in the minigenome RNAs between the S and L segments resulted in altered control of viral protein levels at the posttranscription level (17). We previously used the finding that IGR sequences regulate vmRNA translation efficiency to develop a novel genetic approach for rational attenuation of mammarenaviruses. Recombinant LCMV and LASV containing the S segment IGR (S-IGR) in both the S and L segments were severely attenuated *in vivo* and able to elicit protective immunity against a lethal challenge with their parent virulent strains (18, 19). In addition, partial deletions in the L segment IGRs (L-IGRs) of other mammarenaviruses, Lujo virus and Machupo virus (the causative agent of Bolivian hemorrhagic fever), were associated with an attenuated phenotype in cultured cells (20) and *in vivo* (21). Although the IGR plays a critical role in mammarenaviral multiplication, limited information on the roles of specific vmRNA 3′-UTR regions in controlling translation efficiency has hampered a detailed understanding of the mechanism underlying attenuation by the modified IGR. In this study, we further investigated the contribution of the 3′-UTR sequence of LCMV mRNA to translation regulation employing a reporter system using *in vitro* transcribed mRNA that mimics 5′-capped and 3′-nonpolyadenylated vmRNA. We demonstrate that the 3′-UTR of NP mRNA with no poly(A) tail promotes translation in a poly(A)-binding protein (PABP)-independent manner. We show that a 10-nucleotide (nt) sequence located on the 5′ side of the 3′ UTR of the NP mRNA is essential for promoting translation. Importantly, the reporter protein level was altered in cells infected with recombinant LCMV in which the 10-nt sequence immediately downstream of the reporter gene was modified. Our findings will enable rational design of the 10-nt sequence to further improve our mammarenavirus LAV candidates and to develop a novel LCMV vector capable of controlling foreign gene expression.

## Results

### The 3′-UTR of NP mRNA promotes translation

**Figure 1:**
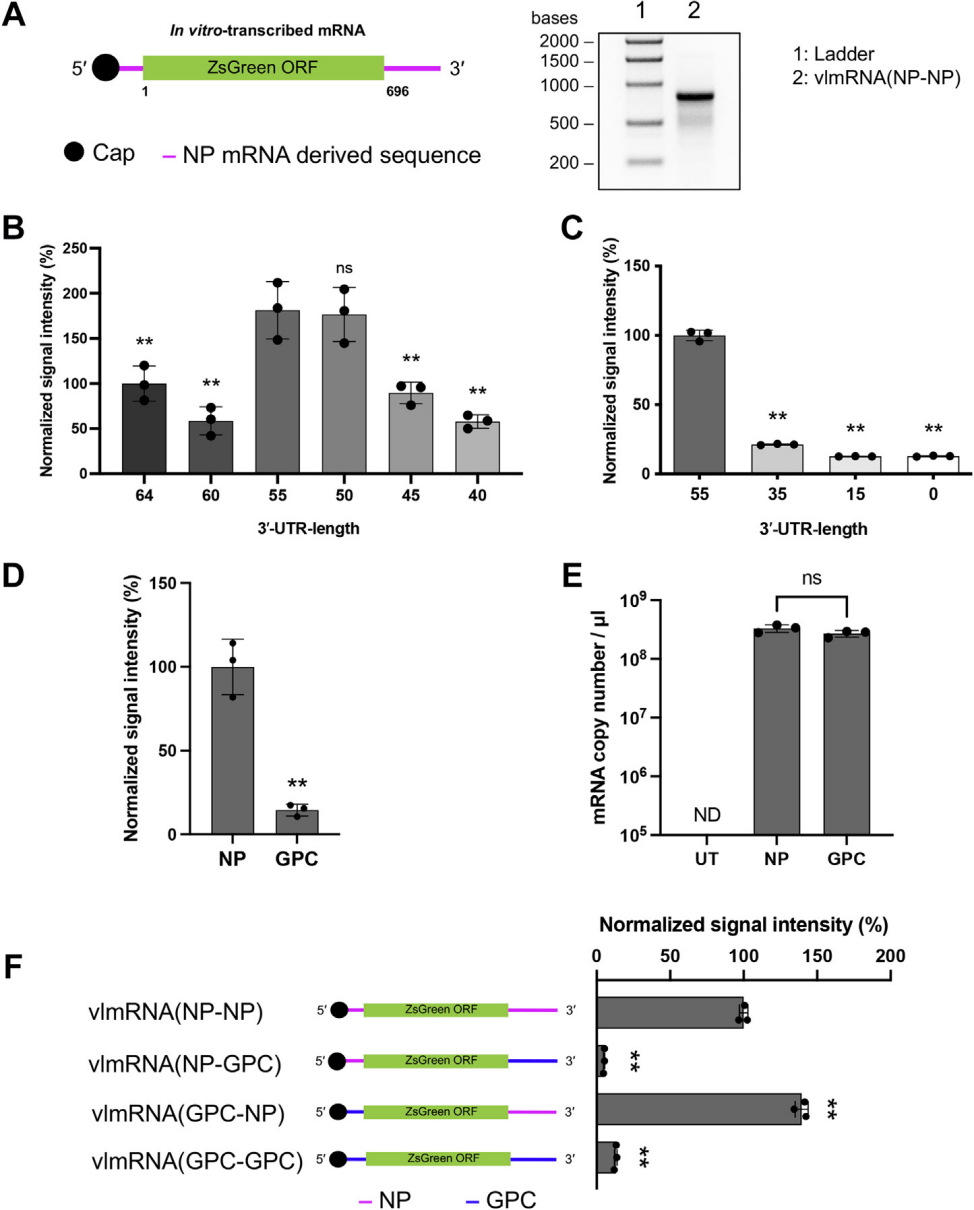
**Contribution of the 5′- and 3′-UTRs of nucleoprotein (NP) and glycoprotein precursor (GPC) mRNAs to translation efficiency.***A*, *left*, schematic diagram of *in vitro* transcribed viral mRNA-like mRNA (vlmRNA) containing 5′- and 3′-UTRs from NP mRNA. ORF, open reading frame. *Right*, agarose gel electrophoresis of an *in vitro* transcribed vlmRNA containing 5′- and 3′-UTRs from NP mRNA [vlmRNA(NP-NP)]. *B* and *C*, translation efficiency of vlmRNA with 3′-UTRs of different lengths. HEK293 cells were transfected with vlmRNA containing the 5′-UTR and serially truncated 3′-UTRs of NP mRNA ranging from 64 nt (the length of the whole S segment intergenic region) to 0 nt. The ZsGreen (ZsG) signal intensity was measured 24 h posttransfection. The mean intensity values for the 64- (*B*) and 55-nt-long (*C*) 3′-UTRs were set to 100%. *D* and *E*, contributions of UTR sequences from NP and GPC mRNAs to translation efficiency. HEK293 cells were transfected with vlmRNA(NP-NP) (NP) or vlmRNA(GPC-GPC) (GPC) or remained untransfected (UT). At 24 h posttransfection, the ZsG signal intensity was measured using a fluorescence plate reader (*D*), and vlmRNA levels in the transfected cells were analyzed by reverse-transcription quantitative PCR (RT-qPCR) (*E*). ND, not detected. *F*, *left*, schematic diagrams of the vlmRNAs. *Magenta* and *blue lines* indicate the UTR sequences from NP mRNA and GPC mRNA, respectively. *Right*, HEK293 cells were transfected with the corresponding vlmRNAs. The ZsG signal intensity was measured using a fluorescence plate reader 24 h postinfection. Data represent the mean and standard deviation of three independent experiments. Statistical significance was determined by comparing the signal intensity values with the value for vlmRNA(NP-NP). ns, no significance; ∗∗ *p* < 0.01. UTR, untranslated region.

Using a minigenome system, we previously demonstrated that IGR sequences play a critical role in vmRNA translation efficiency (17). To further investigate the roles of specific regions in the *cis*-acting regulatory sequence of LCMV mRNA, we used a reporter system with an *in vitro* transcribed vmRNA-like mRNA (vlmRNA) for the fluorescent reporter gene ZsGreen (ZsG), flanked by a capped 5′-UTR and nonpolyadenylated 3′-UTR (Fig. 1*A*). This reporter system allows the accurate assessment of protein levels driven by the UTR sequences of vmRNA without being affected by viral transcription and replication activities. Intriguingly, the 3′ end of the LCMV NP mRNA has been reported to have a high degree of heterogeneity (12). To examine whether the length of the 3′-UTR of LCMV NP mRNA plays a critical role in translation efficiency, we assessed ZsG levels in cells transfected with a series of 3′-end-truncated vlmRNAs. We generated vlmRNAs using PCR fragments containing the 3′-UTR that were serially truncated from 64 nt (full length of the S-IGR) as templates for *in vitro* transcription. Among the vlmRNAs with 3′-UTRs 64 to 40 nt long, the ZsG levels (signal intensities) were highest in HEK293 cells that were transfected with vlmRNAs that had 55- and 50-nt-long 3′-UTRs (Fig. 1*B*). Further truncation did not improve the ZsG levels (Fig. 1*C*). These results indicate that LCMV NP mRNAs with 3′-UTRs 50 to 55 nt long exhibit better translation efficiency among NP mRNAs with variable lengths of 3′-UTR. We used a 55-nt-long 3′-UTR as the representative 3′-UTR of LCMV NP vmRNA for the subsequent experiments.

In contrast to the significant heterogeneity of the 3′ ends of NP mRNA, the 3′ ends of GPC mRNA formed a cluster at the distal side of the IGR (12). Accordingly, for the GPC vlmRNA, we used the 53-nt-long 3′-UTR that was the longest among clones of PCR fragments containing the 3′-UTR of LCMV GPC mRNA because we considered that it would contain all of the functional domains involved in translation regulation. We generated vlmRNAs containing 5′- and 3′-UTRs of NP mRNA [vlmRNA(NP-NP)] or GPC mRNA [vlmRNA(GPC-GPC)] and assessed the effects of the viral 5′- and 3′-UTR sequences on protein levels and RNA stability in the transfected cells. The signal intensity of ZsG in the cells transfected with vlmRNA(GPC-GPC) was approximately seven times lower than that in cells transfected with vlmRNA(NP-NP) without significant reduction of the vlmRNA level (Fig. 1, *D* and *E*), which indicates that production of viral protein is regulated by the UTRs of vmRNA at the translational level.

In a previous study, we used an LCMV minigenome system and demonstrated that the vmRNA 3′-UTR, not the 5′-UTR, played a critical role in controlling viral protein levels (17). We aimed to reproduce these previous observations in our reporter system by generating a series of vlmRNAs with viral 5′- and 3′-UTRs swapped between the NP and GPC mRNAs. As expected, the ZsG levels were significantly reduced when the 3′-UTR of vlmRNA(NP-NP) was replaced with the 3′-UTR of GPC mRNA [vlmRNA(NP-GPC)] and significantly increased when the 3′-UTR of vlmRNA(GPC-GPC) was replaced with the 3′-UTR of NP mRNA [vlmRNA(GPC-NP)] (Fig. 1*F*).

### PABP is not involved in the vmRNA translation

**Figure 2:**
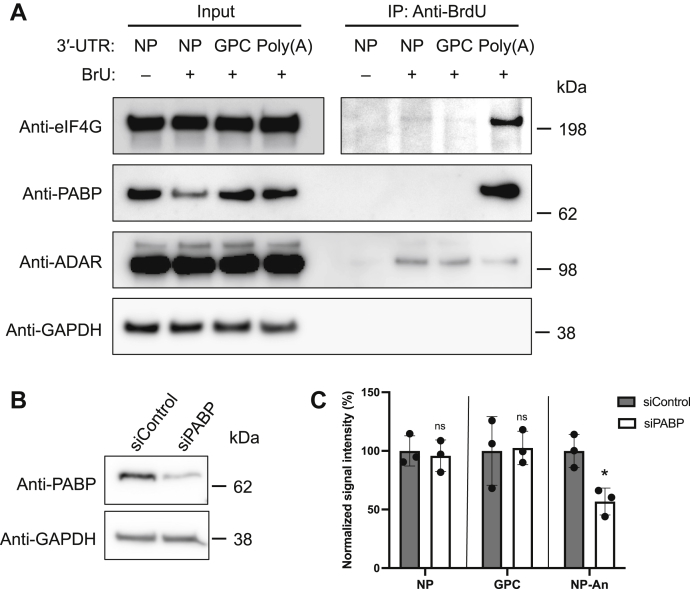
**Assessment of viral mRNA-like mRNA (vlmRNA) binding to host proteins involved in poly(A)-dependent mRNA translation.***A*, HEK293 cells were transfected with vlmRNA(NP-NP) (3′-UTR: nucleoprotein, NP), vlmRNA(NP-GPC) (3′-UTR: glycoprotein precursor, GPC), or vlmRNA(NP-NPAn) [3′-UTR: poly(A)] containing 5-bromouridine (BrU) (BrU: +). vlmRNA(NP-NP) (no BrU incorporation, BrU: −) was transfected into HEK293 cells as a negative control. At 5 h posttransfection, we prepared total cell lysates for RNA-immunoprecipitation assays using an agarose resin coated with an anti-5-bromo-2′-deoxyuridine (BrdU) antibody. Protein levels of eukaryotic initiation factor 4G (eIF4G), poly(A)-binding protein (PABP), adenosine deaminases acting on RNA (ADAR), and GAPDH in cell lysates (Input) and anti-BrdU immunoprecipitates (IP: Anti-BrdU) were analyzed by Western blotting. *B*, reduction of PABP levels by siRNA-mediated gene knockdown (KD). HEK293 cells were transfected with siRNA against PABP (siPABP) or with nonsilencing mismatch siRNA (siControl). At 48 h posttransfection, total cell lysate was prepared, and the protein levels of PABP and GAPDH in cell lysates were determined by Western blotting. *C*, effect of siRNA-mediated KD of PABP on vlmRNA translation. HEK293 cells transfected with either siPABP or siControl for 48 h were further transfected with vlmRNA(NP-NP), vlmRNA(NP-GPC), or vlmRNA(NP-NPAn). At 24 h posttransfection with vlmRNAs, the ZsG signal intensity was measured by a fluorescence plate reader. The mean value for the cells transfected with siControl for each vlmRNA was set to 100%. Data represent the mean and standard deviation of three independent experiments. Statistical significance was determined by comparing the signal intensity values with the value for the cells transfected with siControl. ns, no significance; ∗*p* < 0.05.

Unlike the nonpolyadenylated mRNA of rotavirus, which requires a nonstructural rotavirus protein, NSP3, to promote translation (22, 23, 24, 25), our *in vitro* transcribed vlmRNA reporter system demonstrated that translation could be executed in the absence of viral proteins, suggesting that vmRNA translation is mediated by viral protein-independent cellular machinery. PABP interacts with the poly(A) tail of cellular mRNA and eukaryotic initiation factor 4G (eIF4G), which results in the formation of circular mRNA and facilitates repetitive use of a released ribosome for efficient translation (26, 27, 28). To gain insight into vmRNA translation, we next investigated whether, similar to cellular mRNAs, vmRNA also associated with PABP and eIF4G to promote translation. We generated vlmRNAs containing the 5′-UTR of NP mRNA and the 3′-UTR of NP or GPC mRNA incorporating 5-bromouridine (BrU). A portion of vlmRNA(NP-NP) incorporating BrU was polyadenylated [vlmRNA(NP-NPAn)]. A vlmRNA(NP-NP) lacking BrU was used as a negative control. HEK293 cells were then transfected with the vlmRNAs. At 5 h posttransfection, we prepared total cell lysates for RNA-immunoprecipitation (RIP) assays using an agarose resin coated with an anti-5-bromo-2′-deoxyuridine (BrdU) antibody that cross-reacts with BrU (Fig. 2*A*). PABP was efficiently coimmunoprecipitated with vlmRNA(NP-NPAn), but not with vlmRNA(NP-NP) or vlmRNA(NP-GPC). Likewise, eIF4G was barely detected at similar levels by Western blotting of the anti-BrdU antibody immunoprecipitates of vlmRNA(NP-NP) and vlmRNA(NP-GPC), and eIF4G protein levels were significantly lower than that of vlmRNA(NP-NPAn). To rule out the possibility that different efficiency of coimmunoprecipitation of PABP and eIF4G was due to inconsistent immunoprecipitation efficiency, we also examined the levels of adenosine deaminases acting on RNA (ADAR), which is known to bind to double-strand RNA (29). As we expected, ADAR levels were compatible among anti-BrdU immunoprecipitates of BrU-incorporated vlmRNAs that form a double-stranded stem structure at the 3′-UTR. To further investigate the role of PABP in LCMV mRNA translation, we examined the impact of siRNA-mediated knockdown (KD) of PABP on the translation of vlmRNAs with or without poly(A) (Fig. 2*B*). KD of PABP significantly reduced ZsG levels in cells transfected with vlmRNA(NP-NPAn), but not with vlmRNA(NP-NP) or vlmRNA(GPC-GPC) (Fig. 2*C*). These results indicate that vmRNA translation is regulated in a PABP-independent manner.

### A short 3′-UTR sequence located at the 5′ side of vmRNA is a major determinant of translation efficiency

**Figure 3:**
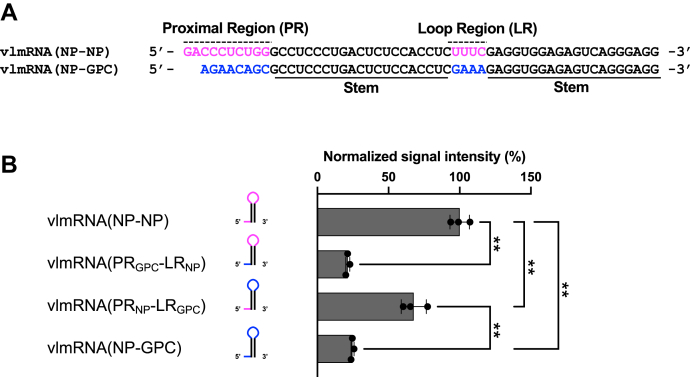
**Role of specific 3′-UTR sequences in translation of viral mRNA-like mRNAs (vlmRNAs).***A*, alignment of the 3′-UTR sequences of vlmRNA(NP-NP) and vlmRNA(NP-GPC). *Magenta* and *blue* indicate vlmRNA(NP-NP) and vlmRNA(NP-GPC) specific regions, respectively; *black lines* indicate the perfectly complementary 21 base pairs that form the stem regions. *B*, *left*, schematic diagrams of the 3′-UTRs of the vlmRNAs. The *magenta* and *blue lines* in the stem-loop structures indicate 3′-UTR sequences specific to the nucleoprotein (NP) and glycoprotein precursor (GPC) mRNAs, respectively; *black lines* represent the stem region. *Right*, translation efficiency of the vlmRNAs containing chimeric 3′-UTRs. HEK293 cells were transfected with the corresponding vlmRNAs. The ZsGreen signal intensity was measured using a fluorescence plate reader 24 h postinfection. The mean value for vlmRNA(NP-NP) was set to 100%. Data represent the mean and standard deviation of three independent experiments. Statistical significance was analyzed by one-way ANOVA, and statistically significant differences were determined by Tukey’s multiple comparisons test. ∗∗*p* < 0.01. UTR, untranslated region.

The LCMV S-IGR contains a putative hairpin structure with a 21-nt paired stem. Because of the perfect complementarity of the stem region, the 3′-UTR sequences derived from the stem region of the NP and GPC mRNAs were identical. In addition, we used 55-nt- and 53-nt-long 3′-UTRs as representative 3′-UTRs of NP and GPC mRNAs, respectively, resulting in generation of the same 3′-termini. Therefore, the 3′-UTR unique to vlmRNA(NP-NP) and vlmRNA(NP-GPC) can be limited to the region located upstream of the stem region (proximal region, PR) and loop region (LR) within the putative hairpin structure (Fig. 3*A*). To examine the contributions of the PR and LR sequences to translation regulation, we generated vlmRNAs with the 5′-UTR of NP mRNA containing chimeric 3′-UTRs with the PR and LR from NP mRNA and GPC mRNA in all possible combinations. Replacing the PR of vlmRNA(NP-NP) with that of GPC mRNA [vlmRNA(PR_GPC_-LR_NP_)] decreased the ZsG level to a value similar to that for vlmRNA(NP-GPC) (Fig. 3*B*). Conversely, replacement of the PR of vlmRNA(NP-GPC) with that of NP mRNA [vlmRNA(PR_NP_-LR_GPC_)] significantly increased the ZsG level to a value that was moderately lower (32%) than that of vlmRNA(NP-NP). These results indicate that the PR sequence of vmRNA is essential for translation regulation, whereas the LR sequence only partially contributes to vmRNA translation.

### Sequence specificity of the PR to promote translation

**Figure 4:**
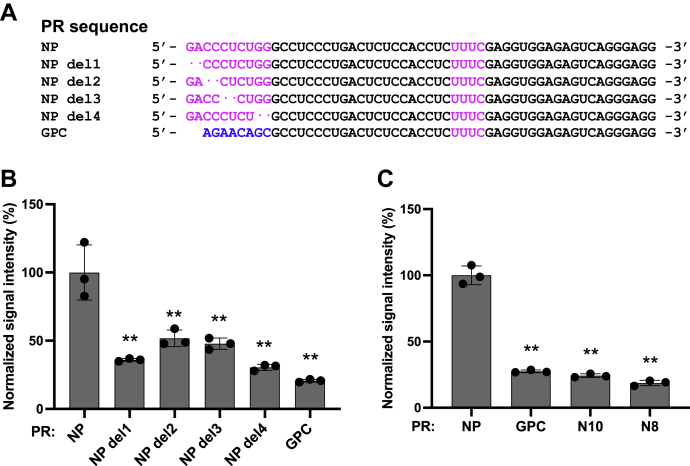
**Contribution of the length of the proximal region (PR) in the 3′-UTR of nucleoprotein (NP) mRNA to translation efficiency.***A*, alignment of the 3′-UTR sequences of vlmRNA(NP-NP)-deletion mutants. *Magenta* and *blue* indicate NP and glycoprotein precursor (GPC) mRNA-specific bases, respectively. *B*, translation efficiency of the vlmRNA(NP-NP)-deletion mutants. HEK293 cells were transfected with the indicated viral mRNA-like mRNAs (vlmRNAs). The ZsGreen (ZsG) signal intensity was measured using a fluorescence plate reader 24 h post-infection. *C*, contribution of the PR length to translation efficiency. The ZsG signal intensities of HEK293 cells transfected with vlmRNA containing random 10-nucleotide (nt) (PR: N10) or 8-nt (PR: N8) sequences in the PR are compared with those of cells transfected with vlmRNA(NP-NP) (PR: NP) or vlmRNA(PR_GPC_-LR_NP_) (PR: GPC). NP del1–del4, vlmRNA(NP-NP)-deletion mutants. The mean value for vlmRNA(NP-NP) (PR: NP) was set to 100%. Data represent the mean and standard deviation of three independent experiments. Statistical significance was determined by comparison with the values for vlmRNA(NP-NP). ∗∗*p* < 0.01.

To identify the PR sequence that promotes translation, we generated vlmRNA(NP-NP)-deletion mutants with 2-nt deletions in the PR. Because the 10-nt PR of the NP mRNA contains a CUCU sequence, the 2-nt deletions within the PR resulted in four vlmRNA(NP-NP)-deletion mutants (del1–del4) (Fig. 4*A*). The ZsG levels were significantly decreased in cells transfected with these vlmRNA(NP-NP)-deletion mutants, although the reductions varied from 48% to 70% (Fig. 4*B*), which indicates that the absence of any of the 2-nt pairs reduced the translation efficiency of NP mRNA. This result and the finding that the PRs of the NP and GPC mRNAs were 10 and 8 nt long, respectively, led us to explore the possibility that the PR length was important for translation regulation. For this, we examined ZsG levels in cells transfected with vlmRNA(NP-NP) containing random 10-nt or 8-nt sequences instead of the actual PR sequence of NP mRNA. The ZsG levels in cells transfected with the two vlmRNA(NP-NP) mutants with the random 10-nt or 8-nt sequences as well as those with vlmRNA(NP-GPC) were significantly reduced when compared with those of the cells transfected with vlmRNA(NP-NP), which indicates that the PR length was not a sufficient condition to promote translation (Fig. 4*C*).

### Involvement of the secondary structure of the PR in regulating translation efficiency

**Figure 5:**
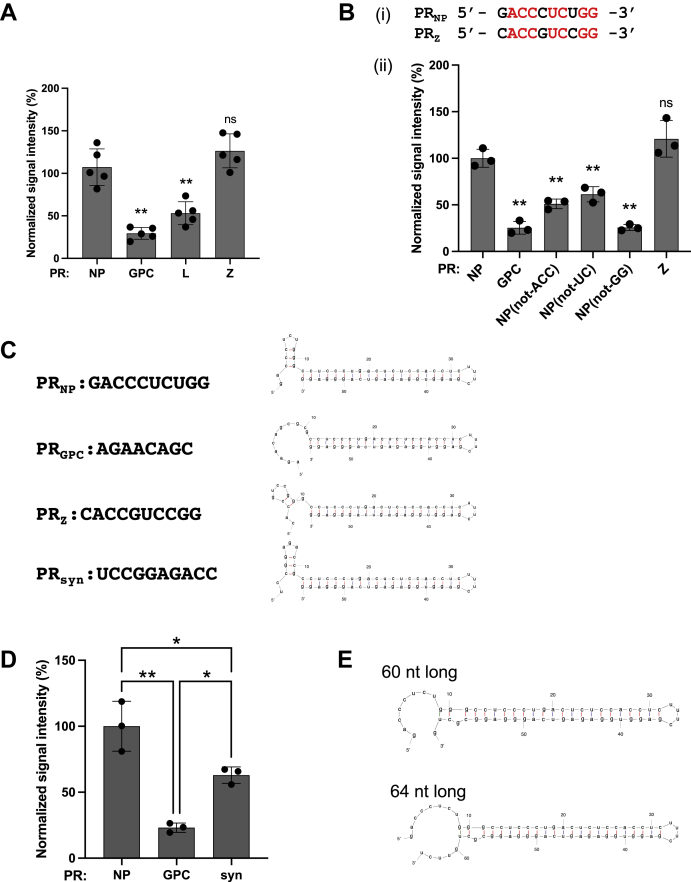
**Involvement of the proximal region (PR) secondary structure of the 3′-UTR in translation efficiency.***A*, ability of PR sequences from L and Z mRNAs to support translation in the context of nucleoprotein (NP) mRNA. HEK293 cells were transfected with vlmRNA(NP-NP) (PR: NP) or viral mRNA-like mRNA (vlmRNA) mutants where the PR sequence of vlmRNA(NP-NP) was replaced with that of glycoprotein precursor (GPC) (PR: GPC), L (PR: L), or Z (PR: Z) mRNA. The ZsGreen (ZsG) signal intensity was measured using a fluorescence plate reader 24 h postinfection. *B*, effects of incorporating substitutions into common bases between the NP and Z PR sequences. i, alignment of the PR sequences of NP and Z mRNA. Red indicates the common bases between NP and Z PR sequences. ii, in addition to vlmRNA(NP-NP) (PR: NP) and its mutants, PR: GPC and PR: Z shown in *A*, other vlmRNA(NP-NP) mutants containing substitutions in the common bases [PR: NP(not-ACC), PR: NP(not-UC), and PR: NP(not-GG)] were transfected into HEK293 cells. The ZsG signal intensity was measured using a fluorescence plate reader 24 h posttransfection. *C*, engineering of artificial PR sequences. PR sequences and putative secondary structures of the 3′-UTRs of vlmRNA(NP-NP), vlmRNA(PR_GPC_-LR_NP_), vlmRNA(PR_Z_-LR_NP_), and vlmRNA(PRsyn-LR_NP_), which contains an artificial PR sequence (PRsyn) that was predicted to form a small hairpin structure similar to that of PR_NP_, are shown. *D*, translation efficiency of vlmRNA(PRsyn-LR_NP_). The ZsG signal intensities of HEK293 cells transfected with vlmRNA(NP-NP) (PR: NP), vlmRNA(PR_GPC_-LR_NP_) (PR: GPC), and vlmRNA(PRsyn-LR_NP_) (PR: syn) are shown. *E*, 3′-UTR sequences longer than 55 nt do not form a putative small stem loop structure at the PR. Predicted secondary structures of 60- and 64-nt-long NP mRNA 3′-UTRs are shown. The mean value for vlmRNA(NP-NP) (PR: NP) was set to 100%. Data represent the mean and standard deviation of five (*A*) or three (*B* and *D*) independent experiments. Statistical significance was analyzed by one-way ANOVA, and statistically significant differences were determined by Dunnett’s multiple comparisons test in comparison with the values for vlmRNA(NP-NP) (*A* and *B*) or by Tukey’s multiple comparisons test (*D*). ns, no significance; ∗*p* < 0.05; ∗∗*p* < 0.01. UTR, untranslated region.

The 3′-UTRs of the L and Z mRNAs transcribed from L segment RNA also play a critical role in translation regulation (17). To examine this further, we determined whether the PR sequence from L or Z mRNA also supported low or high translation efficiency, respectively, in the context of the NP mRNA 3′-UTR. For this, we replaced the PR sequence of vlmRNA(NP-NP) with an 8-nt or 10-nt sequence just downstream of the translation termination codon of L mRNA (low translation efficiency) [vlmRNA(PR_L_-LR_NP_)] or Z mRNA (high translation efficiency) [vlmRNA(PR_Z_-LR_NP_)], respectively. vlmRNA(PR_Z_-LR_NP_) drove efficient production of ZsG equal to that with vlmRNA(NP-NP) (Fig. 5*A*). Conversely, the ZsG levels in cells transfected with vlmRNA(PR_L_-LR_NP_) were significantly reduced compared with the level with vlmRNA(NP-NP) and similar to that with vlmRNA(PR_GPC_-LR_NP_). Intriguingly, the PR sequence of Z mRNA (PR_Z_) had a high degree of sequence identity with that of NP mRNA (PR_NP_), where seven of the 10 nts were identical (Fig. 5*B*, i). To identify nt bases in PR_Z_ that were identical to those in PR_NP_ required for efficient translation of vlmRNA(NP-NP), we introduced nt substitutions so that the newly generated vlmRNA did not contain the original nt bases in the three parts of PR_NP_, ACC, UC, and GG, identical to PR_Z_, of vlmRNA(NP-NP). The resulting vlmRNAs both showed significant reductions in ZsG levels, but to different degrees (39%–74%) (Fig. 5*B*, ii).

Because we failed to clearly identify specific nt bases in the PR_NP_ critical for NP mRNA translation, we considered whether the secondary structure, rather than the primary sequence, was involved in translation regulation by PR_NP_. For this, we compared the predicted secondary structures of the 3′-UTRs of vlmRNA(NP-NP) and vlmRNA(PR_GPC_-LR_NP_) using MFOLD (30). We found a small hairpin structure in PR_NP_ (Fig. 5*C*) that was not found in the PR_GPC_ secondary structure. Intriguingly, the predicted structure of the 3′-UTR of vlmRNA(PR_Z_-LR_NP_) also contained a small hairpin structure, but its base-paired stem was shorter than that of vlmRNA(NP-NP). To examine whether the small hairpin structure contributed to NP mRNA translation, we designed a PR sequence (synthetic PR_NP_-structure-forming sequence, PRsyn) that formed a small stem-loop structure that mimicked the small hairpin found in PR_NP_ but contained drastic (nine out of 10 nts) substitutions in the PR of vlmRNA(NP-NP) (Fig. 5*C*). The ZsG levels were retained at approximately 60% in cells transfected with vlmRNA(PRsyn-LR_NP_) compared with the ZsG levels in cells transfected with vlmRNA(NP-NP) (Fig. 5*D*). However, the PR sequence was largely changed, having substitutions in positions where substitutions led to impaired translation efficiency equivalent to vlmRNA(PR_GPC_-LR_NP_) (*e.g.*, substitutions in GG residues in PR_NP_, Fig. 5*B*). These results indicate that, in addition to the primary sequence, the small hairpin structure in the PR of NP mRNA may play an important role in translation. Consistent with this, the 60- or 64-nt-long 3′-UTR of NP mRNA, with which translation efficiency of vlmRNA was significantly reduced compared with vlmRNA(NP-NP), was not predicted to form the small stem loop structure at the PR (Fig. 5*E*).

### Modifying the IGR sequence corresponding to the PR of recombinant LCMV resulted in altered expression of its upstream gene in virus-infected cells

**Figure 6:**
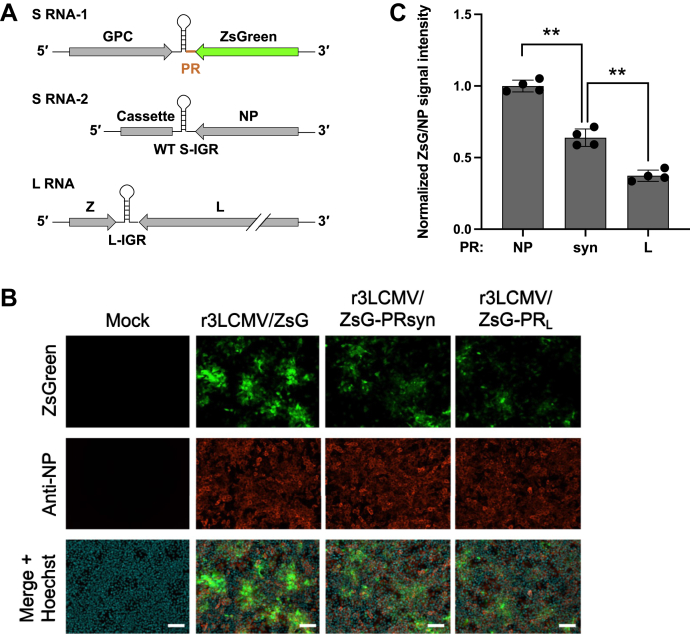
**Effect of modifications in the S segment intergenic region (S-IGR) sequence corresponding to the proximal region (PR) sequence on viral protein expression.***A*, schematic diagram of the genome organization of recombinant trisegmented lymphocytic choriomeningitis virus (r3LCMV) expressing ZsGreen (ZsG) (r3LCMV/ZsG). The *orange line* in the S-IGR corresponding to the PR of ZsG indicates the region modified to generate r3LCMV/ZsG mutants. WT, wild type; Cassette, a gene cassette sequence that does not contain open reading frames. *B* and *C*, altered ZsG levels by modification of the PR. Vero E6 cells were infected (multiplicity of infection = 0.1) with r3LCMV/ZsG or r3LCMV/ZsG mutants where the PR_NP_ sequence downstream of the ZsG gene was replaced with PR_L_ (r3LCMV/ZsG-PR_L_) or PRsyn (r3LCMV/ZsG-PRsyn). At 48 h postinfection, the cells were fixed, and nucleoprotein (NP) was detected by indirect immunofluorescence using the anti-NP antibody VL-4 (*red*). Nuclei were visualized by Hoechst 33342 (Hoechst) staining (*blue*). Stained cells were observed using a confocal microscope (*B*). Scale bars = 100 μm. ZsG and NP signal intensities were measured using a fluorescence plate reader (*C*). The mean signal intensity value of ZsG normalized to that of NP for r3LCMV/ZsG was set to 1. Data represent the mean and standard deviation of four independent experiments. Statistical significance was determined by comparison with the values for r3LCMV/ZsG-PRsyn. ∗∗*p* < 0.01.

The results from our reporter system indicated that the PR in NP and GPC mRNAs is a major determinant of translation efficiency. To validate our findings at the virus level, we took advantage of a recombinant trisegmented LCMV (r3LCMV) platform (31, 32). The r3LCMV platform developed by separating two essential genes, NP and GPC, into two different S segments provides two additional transcriptional units into which one or two genes of interest can be inserted. With this approach, we generated r3LCMV, where the ZsG sequence was inserted into the NP locus on the opposite side of the GPC gene (r3LCMV/ZsG) (Fig. 6*A*). To investigate the impact of manipulation of the PR on the expression of its upstream gene in virus-infected cells, the PR sequence of r3LCMV/ZsG was replaced with that of PRsyn (moderately less translation efficiency than PR_NP_) or PR_L_ (low translation efficiency, similar to PR_GPC_), which resulted in the generation of r3LCMV/ZsG-PRsyn and r3LCMV/ZsG-PR_L_, respectively. We inoculated these three r3LCMVs into Vero E6 cells and determined the ZsG and NP levels in the virus-infected cells 48 h postinfection. Although the ZsG levels varied from cell to cell, the overall ZsG levels, consistent with the results from our reporter system, appeared to be decreased when the PR sequence of r3LCMV/ZsG was replaced with that of PRsyn and decreased further with PR_L_, with no apparent differences in NP levels (Fig. 6*B*). Quantitative analysis by measuring the ZsG signal intensity of each well using a fluorescence plate reader confirmed the stepwise decrease in overall ZsG levels by the replacement of the PR sequence (Fig. 6*C*).

## Discussion

Asymmetric NP and GPC levels are well documented, and NP production has been shown to be significantly higher than GPC production in LCMV-infected cells. This expression balance is critical for LCMV because abrogation of the balance by replacing the S-IGR with the L-IGR resulted in nonviable virus (17). Similar to that of other negative-strand RNA viruses, mammarenaviral polymerase generates significantly higher levels of genomic RNA species than the complementary antigenomic RNA species (17, 33). Because of the ambisense coding strategy, by which the viral polymerase uses the same promotor for both viral genome replication and gene transcription, mammarenaviruses cannot balance viral protein levels by controlling mRNA levels (11). Mammarenaviral regulation of protein levels by nonpolyadenylated 3′-UTR sequences, not by amounts, may help to overcome this constraint (17). Despite the critical role in mammarenaviral viability, only limited information about the 3′ ends of vmRNAs is currently available. One factor that has hampered investigation of the 3′-UTRs of vmRNAs is the limited availability of sophisticated techniques to separate genome RNA species and nonpolyadenylated vmRNA species. Meyer and Southern (12) mapped the 3′ ends of LCMV NP and GPC mRNAs by sequencing PCR fragments containing regions that corresponded to the 3′–5′ junction of circularized vmRNA isolated by CsCl density-gradient centrifugation. Intriguingly, the 3′ ends of the NP mRNA exhibited a high degree of heterogeneity. Conversely, our results indicate that the optimal length of the 3′-UTR of the NP mRNA for translation is 50 to 55 nt and that vlmRNAs with 3′-UTRs shorter than 45 nt significantly reduced ZsG levels when compared with vlmRNA(NP-NP). A possible explanation for this discrepancy is that the structured 3′-termini of vmRNA prevented efficient ligation with their 5′-termini, causing biased variations of circularized vmRNAs. Furthermore, no information about the 3′ ends of LCMV L and Z mRNAs is currently available. Our results show that PR_L_ reduced and PR_Z_ equally promoted translation, when compared with PR_NP_, in the context of the 3′-UTR of NP mRNA. However, whether PR_L_ and PR_Z_ also contribute to the regulation of L and Z protein levels in LCMV-infected cells remains unclear. Further precise mapping of the 3′ ends of all four LCMV mRNAs using a state-of-the-art sequencing technique to read the 3′ ends of nonpolyadenylated RNAs (*e.g.*, long noncoding RNAs) may help to elucidate these issues.

The genomic RNA species of dengue virus encodes a viral polyprotein. The coding region is flanked by 5′-capped and 3′-nonpolyadenylated UTRs, and dengue virus genomic RNA serves as an mRNA in infected cells. The highly structured 3′-UTR of the dengue virus genomic RNA contains a 3′ stem-loop and two dumb-bell structures and binds to PABP through the A-rich regions that flank the two dumb-bell structures upstream of the 3′ stem-loop (34). Conversely, a Bunyavirus family virus, Bunyamwera virus (BUNV), which also produces mRNA species with 5′-capped and 3′-nonpolyadenylated UTRs, executes translation in a PABP-independent manner (35), which indicates that an as-yet-unidentified host protein may bridge the 5′ and 3′ ends to promote the formation of circular mRNA. Intriguingly, translation of BUNV mRNA requires a region located at the 3′ end that forms a stem-loop structure. In the present study, we identified a small region, PR_NP_, that significantly enhanced translation efficiency and was also predicted to form a small stem-loop structure. In addition, our RIP assays resulted in no clear binding of vlmRNA to PABP. These findings support the hypothesis that the small stem-loop structure present in PR_NP_ enhanced the translation efficiency of viral proteins, potentially by recruiting cellular proteins other than PABP required for the circularization of vmRNA.

We demonstrated, by RT-qPCR, that vlmRNA levels were compatible between cells transfected with vlmRNA(NP-NP) and those with vlmRNA(GPC-GPC). Because the template total RNA for RT-qPCR might contain biologically active vlmRNA and vlmRNA retained with the transfection reagent and not released into cell cytoplasm, comparison of vlmRNA levels by RT-qPCR could underestimate the differences in biologically active vlmRNA levels. Of note, we previously showed, using an LCMV minigenome system, that reporter protein levels were significantly altered by the replacement of the IGRs between S and L segments without significantly impacting on intracellularly expressed reporter mRNA levels, suggesting that 3′-UTR sequences of LCMV mRNA do not significantly affect the stability of mRNA (17). Meanwhile, polysome analysis is a powerful system to investigate biologically active mRNAs. Aviner *et al.* (36) recently revealed, using the combination of polysome analysis and mass spectrometry analysis, that flaviviruses modify polysome compositions by evicting or recruiting several types of host cell machinery for their preferred biogenesis. Polysome analysis of LCMV-infected cells and identification of host cell factors incorporated in polysomes should help to elucidate the mechanism of translation regulation by the 3′-UTR of LCMV mRNA.

Because LCMV can induce robust long-term CD8+ cytotoxic T lymphocyte responses against virus antigens, attenuated recombinant LCMV has been proposed as a tumor immunotherapy platform to deliver tumor-associated antigens (37). Our results indicate that the expression of foreign genes from r3LCMV can be altered by modification of the PR sequence. This finding provides opportunities to improve recombinant LCMV-based gene delivery systems to fine-tune the expression of a foreign gene as well as the degree of attenuation by altering the PR sequence. In addition to the noncytolytic nature and broad tissue tropism, this flexibility to manage foreign gene expression levels expands the utility of a recombinant LCMV-based gene delivery system to more demanding applications, such as the generation of induced pluripotent stem cells, in which the levels of transduced reprogramming factors must be precisely controlled for effective derivation (38, 39).

## Experimental procedures

### Plasmids

Plasmids expressing LCMV NP (pC-NP) and LCMV L (pC-L) have been described previously (40). Plasmids for r3LCMV generation were constructed based on the mPol1Sag and mPol1Lag plasmids that direct RNA polymerase I (Pol1)-mediated intracellular synthesis of the S and L RNA antigenome species of the Clone 13 strain of LCMV (32, 41). mPol1Sag(BbsI/GPC) and mPol1Sag(NP/BsmBI) containing gene cassette sequences in the NP and GPC loci for the insertion of genes of interest using restriction enzyme sites, *Bbs*I or *Bsm*BI, respectively, have been described previously (32). To generate mPol1Sag(ZsG/GPC), a DNA fragment containing the ZsG open reading frame was inserted into the NP locus of mPol1Sag(BbsI/GPC) using the *Bbs*I site. mPol1Sag(ZsG-PRsyn/GPC) and mPol1Sag(ZsG-PR_L_/GPC) were generated by PCR-based mutagenesis replacing the PR sequence for ZsG in mPol1Sag(ZsG/GPC) with PRsyn and PR_L_, respectively.

### Cells

HEK293 (American Type Culture Collection, ATCC, CRL-1573), Vero E6 (ATCC, CRL-1586), and BHK-21 (ATCC, CCL-10) cells were grown in Dulbecco’s modified Eagle medium (DMEM; Nacalai Tesque) containing 10% heat-inactivated fetal bovine serum, 100 U/ml penicillin, and 100 μg/ml streptomycin at 37 °C and 5% CO_2_.

### *In vitro* synthesis of vlmRNA

DNA fragments composed of the T7 promoter sequence with a GGG sequence at the 3′ end, 5′-UTR sequence, ZsG open reading frame with the stop codon, and 3′-UTR sequence in this order were amplified by PCR. These DNA fragments were used as templates to generate 5′-capped and 3′-nonpolyadenylated vlmRNAs using a T7 mScript Standard mRNA Production System (CELLSCRIPT). Transcription by T7 polymerase was initiated at the first G position of the GGG sequence at the 3′ end of the T7 promotor sequence, and therefore vlmRNAs contained a GGG sequence at the 5′-termini. We employed the T7 promoter sequence with the GGG sequence to increase the transcription efficiency of T7 polymerase and to use it as a surrogate for the nontemplated 5′-terminus sequences of mammarenavirus mRNAs derived from cellular mRNA by cap snatching (12, 15). *In vitro* transcribed vlmRNAs were purified using Monarch RNA cleanup kit (New England Biolabs), and the quality of vlmRNAs was assessed by nondenaturing agarose gel electrophoresis using RNA High for Easy Electrophoresis (DynaMarker Laboratory). For RIP assays, 5-bromouridine 5′-triphosphate was added to *in vitro* transcription reactions to generate BrU-containing vlmRNAs. A part of the BrU-containing vlmRNA(NP-NP) was polyadenylated using the T7 mScript Standard mRNA Production System (CELLSCRIPT). To incorporate mutations in the 5′-UTRs and 3′-UTRs of the vlmRNAs, PCR primers containing corresponding substitutions were used to PCR amplify the DNA templates for *in vitro* transcription of vlmRNA. To generate vlmRNAs that possessed random nt bases (Fig. 4*C*) or nt bases that were not the same as the original bases in the PR (Fig. 5*B*), DNA templates were amplified by PCR using primers containing mixed bases in the corresponding regions.

### Assessment of ZsG expression in cells transfected with vlmRNA

HEK293 cells seeded in 96-well plates at 5 × 10^4^ cells per well and cultured overnight were transfected with 200 ng of vlmRNA using 0.5 μl of Lipofectamine 2000 (Thermo Fisher Scientific). At 5 h posttransfection, the transfection mixture was removed, and fresh medium was added. At 24 h posttransfection, the cells were fixed with 4% paraformaldehyde in PBS (4% PFA/PBS) (Nacalai Tesque), and the ZsG signal intensity was measured using a fluorescent plate reader (Powerscan HT, BioTech).

### RT-qPCR

HEK293 cells seeded in 96-well plates at 5 × 10^4^ cells per well and cultured overnight were transfected with 200 ng of vlmRNA using 0.5 μl of Lipofectamine 2000 or left untransfected. At 5 h posttransfection, the transfection mixture was removed and fresh medium was added. At 24 h posttransfection, total RNA was isolated and reverse-transcribed into cDNA with a random primer using a CellAmp Direct TB Green RT-qPCR Kit (Takara Bio). The amounts of cDNAs for the vlmRNAs were quantified using a CellAmp Direct TB Green RT-qPCR Kit and a qPCR Thermal Cycler (AriaMx, Agilent). The primers used for amplification of the cDNAs containing the ZsG coding region by real-time PCR were as follows: forward primer: 5′-CCCCGTGATGAAGAAGATGA-3′, reverse primer: 5′-GTCAGCTTGTGCTGGATGAA-3′. Copy numbers were determined by a standard curve method.

### RIP assay

HEK293 cells seeded in a 175-cm^2^ flask and cultured overnight were transfected with 60 μg of BrU-containing vlmRNA(NP-NP), vlmRNA(NP-GPC), or vlmRNA(NP-NPAn). Additionally, 60 μg of vlmRNA(NP-NP) (not containing BrU) was used as a negative control. At 5 h posttransfection, total cell lysates were prepared, and vlmRNAs in the clarified cell lysates were immunoprecipitated with an anti-BrdU antibody using a RiboTrap kit [Medical & Biological Laboratories (MBL)], in accordance with the manufacturer's protocol. Clarified cell lysates and precipitates were mixed at a 3:1 ratio with 4 × Bolt LDS Sample Buffer (Thermo Fisher Scientific) supplemented with Bolt Sample Reducing Agent (Thermo Fisher Scientific) and incubated for 10 min at 70 °C. Protein samples were fractionated by sodium dodecyl sulfate–polyacrylamide gel electrophoresis using 4% to 12% gradient polyacrylamide gels (Blot Bis-Tris Plus Gel 4 to 12%; Thermo Fisher Scientific), and the resolved proteins were transferred by electroblotting onto polyvinylidene difluoride membranes (Immobilon-P PVDF Transfer Membranes; Millipore). To detect specific proteins, the membranes were incubated with rabbit polyclonal antibodies to eIF4G1 (RN002P; MBL), ADAR1 (D7E2M; Cell Signaling Technology) and GAPDH (ABS16; Millipore), and a mouse monoclonal antibody to PABPC1 (RN009M; MBL), followed by incubation with horseradish-peroxidase-conjugated anti-rabbit or anti-mouse IgG antibodies as appropriate (Jackson ImmunoResearch Laboratories). The Chemi-Lumi One L or Chemi-Lumi One Ultra chemiluminescent substrate (Nacalai Tesque) was used to generate chemiluminescent signals that were visualized with an ImageQuant LAS 4000 mini biomolecular imager (GE Healthcare Bio-Sciences).

### siRNA-mediated gene knockdown of PABP

Chemically synthesized 21-nucleotide siRNAs against PABP (siPABP) and nonsilencing mismatch siRNA (siControl) were purchased from Sigma-Aldrich (42). HEK293 cells seeded in a 12-well plate at 2.5 × 10^4^ cells per well and cultured overnight were transfected with 50 nM siPABP or siControl using 2 μl of Lipofectamine RNAi MAX Reagent (Invitrogen). At 48 h posttransfection, the cells were used for further experiments.

### Generation of r3LCMVs

The three different r3LCMVs used in this study were generated by reverse genetics as described previously, with minor modifications (32). For the generation of r3LCMV/ZsG, BHK-21 cells seeded at 7 × 10^5^ cells per well (six-well plate) and cultured overnight were transfected with mPol1Sag(ZsG/GPC) (0.8 μg), mPol1Sag(NP/BsmBI) (0.8 μg), and mPol1Lag (1.4 μg) together with pC-NP (0.8 μg) and pC-L (1 μg) using 12 μl of Lipofectamine 2000 and incubated at 37 °C and 5% CO_2_. At 5 h posttransfection, the transfection mixture was removed and fresh medium was added to the well. After 3 days of incubation at 37 °C and 5% CO_2_, the cell culture medium (tissue culture supernatant, TCS) was removed, fresh medium was added to the well, and the plates were then cultured at 37 °C and 5% CO_2_ for another 3 days. The TCS collected at 6 days posttransfection was used to amplify the rescued virus. r3LCMV/ZsG-PRsyn and r3LCMV/ZsG-PR_L_ were generated by reverse genetics with procedures similar to those used to generate r3LCMV/ZsG, using mPol1Sag(ZsG-PRsyn/GPC) and mPol1Sag(ZsG-PR_L_/GPC), respectively, instead of mPol1Sag(ZsG/GPC).

### Virus titration

r3LCMV titers were determined using an immunofocus forming assay as described previously, with minor modifications (43). Vero E6 cells seeded in 96-well plates at 2 × 10^4^ cells per well and cultured overnight were inoculated with tenfold serial dilutions of r3LCMV. After 20 h of incubation at 37 °C and 5% CO_2_, the cells were fixed with 4% PFA/PBS. After cell permeabilization and blocking by treatment with 1% normal goat serum (FUJIFILM Wako Pure Chemical Corporation) in dilution buffer (0.3% TritonX-100 in PBS containing 3% bovine serum albumin), the cells were incubated with a primary antibody against LCMV NP (VL-4, Bio X Cell), followed by a secondary anti-rat IgG antibody conjugated with Alexa Fluor 568 (anti-rat-AF568) (Thermo Fisher Scientific). Virus titers were calculated by multiplying the NP-positive LCMV-focus number counted under a fluorescent microscope (ECLIPSE Ti2-U, Nikon) by the corresponding dilution factor.

### Assessment of ZsG expression in cells infected with r3LCMVs

Vero E6 cells seeded in 96-well plates at 2 × 10^4^ cells per well and cultured overnight were inoculated with r3LCMV (multiplicity of infection = 0.1). After 48 h of incubation at 37 °C and 5% CO_2_, the cells were fixed with 4% PFA/PBS. After cell permeabilization and blocking by treatment with 1% normal goat serum in dilution buffer, the cells were stained with VL-4 and anti-rat-AF568 for NP and with Hoechst 33342 (Nacalai Tesque) to visualize nuclei. The stained cells were observed using a confocal microscope (CQ1, Yokogawa Solution Service Corporation). Signal intensities of ZsG and NP were measured using a fluorescence plate reader (Powerscan HT, BioTech).

### Statistical analysis

GraphPad Prism 9 (GraphPad) was used for all the statistical analyses. Statistical significance was analyzed by one-way ANOVA, and statistically significant differences were determined by Dunnett’s multiple comparisons test unless otherwise indicated (∗ *p* < 0.05, significant; ∗∗ *p* < 0.01, very significant; ns, *p* > 0.05, not significant).

## Data availability

All data are contained within the article.

## Conflict of interest

The authors declare that they have no conflicts of interest regarding the content of this article.
